# Evidence in surgical training – a review

**DOI:** 10.1515/iss-2018-0026

**Published:** 2019-04-22

**Authors:** Tobias Fritz, Niklas Stachel, Benedikt J. Braun

**Affiliations:** Department of Trauma, Hand and Reconstructive Surgery, Saarland University Medical Center, Kirrbergerstr. 1, 66421 Homburg/Saar, Germany; Department of Orthopaedics and Orthopaedic Surgery, Saarland University Medical Center, Homburg/Saar, Germany; Department of Trauma, Hand and Reconstructive Surgery, Saarland University Medical Center, Homburg/Saar, Germany

**Keywords:** education curriculum, E-learning, residency, simulation training, surgical education

## Abstract

The first residency programs for surgical training were introduced in Germany in the late 1880s and adopted in 1889 by William Halsted in the United States [Cameron JL. William Stewart Halsted. Our surgical heritage. Ann Surg 1997;225:445–58.]. Since then, surgical education has evolved from a sheer volume of exposure to structured curricula, and at the moment, due to work time restrictions, surgical education is discussed on an international level. The reported effect of limited working hours on operative case volume has been variable [McKendy KM, Watanabe Y, Lee L, Bilgic E, Enani G, Feldman LS, et al. Perioperative feedback in surgical training: a systematic review. Am J Surg 2017;214:117–26.]. Experienced surgeons fear that residents do not have sufficient exposure to standard procedures. This may reduce the residents’ responsibility for the treatment of the patient and even lead to a reduced autonomy at the end of the residency. Surgical education does not only require learning the technical skills but also human factors as well as interdisciplinary and interprofessional handling. When analyzing international surgical curricula, major differences even between countries of the European Union with more or less strict curricula can be found. Thus far, there is no study that analyzes the educational program of different countries, so there is no evidence which educational system is superior. There is also little evidence to distinguish the good from the average surgeon or the junior surgeons’ progress during his residency training. Although some evaluation tools are already available, the lack of resources of most teaching hospitals often results in not using these tools as long it is not mandatory by a governmental program. Because of decreased working hours, increasing hospital costs, and increasing jurisdictional restrictions, teaching hospitals and teachers will have to change their sentiments and focus on their way of surgical education before governmental regulations will emerge leading to more regulation in surgical education. Some learning tools such as simulation, electronic learning, augmented reality, or virtual reality for a timely, sufficient and up to date surgical education. However, research and evidence for existing and novel learning tools will have to increase in the next years to allow surgical education for the future generation of surgeons around the world.

## Surgical training programs

Most countries provide a curriculum for surgical education, but the programs differ in their structure and time points of assessment. For example, the United Kingdom and the Republic of Ireland use very structured educational programs with repeating assessments for human factors, technical skills, and also medical knowledge using multiple-choice questionnaires [1]. The Irish and British educational programs try to evaluate surgeons for their daily tasks grading them as a professional communicator, a scholar, a collaborator, an advocate, and a manager [1]. Only after completing these steps that a surgeon may proceed with the training program. This allows a highly standardized level for surgical education throughout the country.

In Germany, the official trainer of the institution certifies that a resident has acquired the necessary knowledge and skills for the required surgical procedures for the residents’ logbook. At the end of the residency program, a theoretical exam is administered by the local medical professional committee. A practical test is not performed so the surgical procedural quality is not part of this certification to distinguish the good from the average surgeon. Some trainers and medical directors state that the German surgical education program allows a more individualized training of the residents because the trainer may decide when the resident is ready to perform the procedure. However, this system allows no comparability between residents of different hospitals or even one teaching hospital.

Kneist et al. stated that the number of procedures performed by the residents increased after implementing a transparent logbook, which could be overseen by all attendings and residents. They even stated that the number requested by the logbook could only be achieved using this transparency [2]. Still, the documentation of procedures in the logbook alone will lack of outside quality control during the residency. Furthermore, there is no evidence whether the number requested in the logbook reproduces the skills acquired by the resident [3]. The only study comparing structured and rather unstructured surgical education programs analyzed the Canadian and Swiss educational programs and even stated that providing a more structured surgical program may be advantageous in providing optimal quality of surgical education [4]. However, the authors only compared one teaching hospital of each country in their study [4].

A comparison of these very different designed educational programs should be analyzed for different countries and centers to achieve evidence whether or not a system is advantageous.

## Assessment of operative performance

To validate the curricula in surgical education for their reliability and efficiency assessments, technical skills are required. These assessments analyze the trainees’ performance in the operating room.

McKendy et al. stated that the implementation of specific teaching strategies using a structured framework improved the feedback within the operation room [5]. Models that can be helpful are such as “None-Technical Skills for Surgeons”, “Briefing Intraoperative Teaching, Debriefing”, and the Five-Step Feedback Tool for Surgery: “Set learning objectivities. How did it go? Address concerns. Review learning points. Plan Ahead” [6].

To analyze the intraoperative performance and competence of trainees, different scores have been developed and validated for their reliability, such as the Ottawa Surgical Competency Operating Room Evaluation (O-SCORE), the Operative Rating System (OPRS), or the Zwisch scale (Table 1) [7].

**Table 1:** Scores that help to assess trainees’ surgical skills.

OPRS	O-SCORE	Zwisch scale	
For example, inguinal herniorrhaphy	Scale: 1–5	*Show and tell*	
A1 (*poor*)–A5 (*excellent*)	1 – *Requires complete hands-on guidance*	–	Attending does key portions as the surgeon narrates the case
1. [numeric]Ilioinguinal nerve	5 – *Complete independence*	*Smart help*	
2. Search for indirect hernia	1. [numeric]Preprocedure plan	–	Attending shifts between surgeon on first assist role and coaching for specific skills
3. Mesh insertion	2. Case preparation	*Dumb help*	
4. Knowledge of anatomy	3. Knowledge of specific procedural steps	–	Attending assists and follows the lead of the resident
5. Femoral vein injury	4. Technical performance	–	Coaches regarding polishing and refinement of skills
6. Prevention of complications	5. Visuospatial skills	*No help*	
7. Respect for tissue	6. Postprocedure plan	–	Attending largely provides no unsolicited advice
8. Time and motion	7. Efficiency and flow	–	Monitors progress and patient safety
9. Flow of operation	8. Communication		
10. Overall performance[/numeric]	9. Resident is able to safely perform this procedure independently		
	10. Give at least one specific aspect of procedure done well		
	11. Give at least one specific suggestion for improvement[/numeric]		

The OPRS has already been described in 2005 by Larson et al., which consists of 10-item procedure-specific rating instruments, including technical skills rating, operative decision making, and general items (each scaled from 1 to 5). They validated the scoring systems for surgical procedures in general surgery and also tested the interrater variability [8]. The usage of this score using case-specific technical skill items allows to evaluate residents postoperatively, which may help to identify strengths or weaknesses. It may also be a helpful tool to create transparency between residents (“Why is my colleague allowed to do that procedure and I am not?”) [8].

Gofton et al. developed and validated the O-SCORE to create a tool to assess surgical competence. The O-SCORE consists of 11 items (8 items rated on the five-point competency scale, 1 yes/no question about competency to perform the procedure independently, and 2 open-ended questions for feedback) and the attending who evaluates the resident after finishing the surgical procedure [9]. The authors validated the O-SCORE for orthopedic and general surgery thus far. They also stated that the score was even able to differentiate among senior and junior trainees and also saw an improvement in performance with an increase of level of training, so this score may be a helpful tool to create objective data of the individual residents’ competencies, which also allows to differentiate which procedure can be performed by the resident at the current time point.

George et al. showed that using a one-dimensional global rating scale is reliable and valid to collect accurate data of residents’ intraoperative performance [7]. These data can be acquired using the Zwisch scale with an integrated smartphone-based method. They even hypothesized that these data could be used to quantify the effects of specific educational interventions [7]. The Zwisch scale analyzes the attendings’ and residents’ behavior intraoperatively. It uses four stages of supervision: show and tell, smart help, dumb help, and no help. A resident should be able to perform the procedure without help. The Zwisch model is a possible approach for guiding faculty and resident interaction in the operating room and may help to evaluate a residents’ level of experience and autonomy [10].

To analyze the performance in surgical simulation, an Objective Structured Assessment of Technical Skills (OSATS) has been validated [11]. Learners are assessed in a series of standardized surgical tasks on inanimate models under direct observation. Candidates are scored in a task-specific checklist consisting of 10–30 specific surgical maneuvers and a second global rating form, which includes five to eight surgical behaviors, such as respect for tissues, economy of motion, and appropriate use of assistants. The reliability and validity of this assessment tool are similar to the Objective Structured Clinical Examination and can be used for summative high-stakes evaluation purposes [11]. For laparoscopic skills, the McGill Inanimate System (MISTEL) and the Imperial College Surgical Assessment Device (ICSAD) were developed [11]. MISTEL uses an inanimate box to simulate the generic skills needed in the performance of laparoscopic surgery and is a valid and reliable instrument. The ICSAD tracks hand motion using sensors placed on the trainees’ hand during the performance of a task. These sensors translate movement into a computerized tracing of hand motion, which provides an effective index of technical skill in both laparoscopic and open procedures [11]. Modern technology such as virtual reality (VR) allows a real-time measurement of the trainees’ performance for precision, accuracy, and error rates and may be a helpful tool to create future curricula for surgical education [12].

Another powerful tool in surgical education may be the implementation of a structured perioperative feedback. Postoperative debriefing about the performance and improvement of residents helps to identify strengths and weaknesses of the resident [13]. Although these tools already existed for years, there is still a lack of their usage in daily business. McKendy et al. described existing feedback mechanisms, which could easily be implemented into perioperative surgical education [6], allowing the attendings to address the problems immediately; also, the residents may communicate the amount and quality of teaching provided by their teachers [6].

## Which modules may help to increase the quality of surgical training?

To train surgical competencies, different methods can be used. Theoretical skills can be acquired using books, current research articles, or an increasing amount of electronic learning (E-learning). Technical skills can be acquired using surgical simulation and of course perform operations with supervision in theater. Due to the increasing standards of quality management, the interest and training of human factors skills (e.g. personal, interpersonal, and interprofessional) have evolved in the last years although not implemented in most surgical education programs thus far [14].

### Perioperative feedback

Feedback has been defined more than 30 years ago and it remains one of the most powerful teaching tools in surgical education because, if used correctly, it can provide an objective assessment of the residents’ performance to improve technical skills [13]. McKendy et al. analyzed in a structured review the main categories of perioperative training: observation of teaching behaviors, perceptions of teaching behaviors, and models for delivering structured feedback [6]. The review showed that residents were not satisfied with the amount of the teaching received. They also showed that attendings reported providing more frequent and better quality feedback than residents reported receiving [6]. This means that attendings may overestimate their amount and quality of teaching or residents do not recognize when being taught [6].

Also, residents described effective teachers as staff who were calm and in control, taught with enthusiasm, and remembered what it was like to be a trainee [6].

### E-learning in surgical education

To learn theoretical content besides classic media such as books, scientific papers, and reviews, a growing number of E-learning tools have evolved in the last years. Internet and software-based learning platforms in medical education have gained great popularity. The widespread use of smartphones, tablets, and multimedia platforms presents new ways to deliver evidence-based educational materials [15]. These E-learning tools range from online textbooks to cognitive stimulators and often involve online curricula [15]. Because of technological improvement, a multitude of E-learning tools have evolved in the last years. Maertens et al. analyzed the literature for their effectiveness as an adjunct to surgical education [15]. These learning tools can be implemented into simulation-based training programs for psychomotor skill acquisitions (e.g. tying a surgical knot) [15]. However, as a result of limited time during working hours and changing priorities of the current generation of young surgeons [16], the compliance rate among surgical residents is still low [5], [15]. More resources during working hours and financial promotion could increase the compliance rate to empower the enormous potential for future surgical education programs.

### Technical skills

Surgeons require to acquire motor skills and experience. Charles Mayo stated that “experience can mean making the same mistake over and over again” [1]. Because of increasing expectations of the society and jurisdictional regulations, mistakes are not tolerable, so novel learning tools are necessary to create experience.

Fitts and Posner described a three-stage theory that is widely accepted in the literature [11]. Therefore, the learner has to understand the task by explanation and demonstration, performing erratic and distinct steps. This is followed by deliberate practice and feedback with a more fluid performance and fewer interruptions to achieve an integrative stage, in which knowledge is translated into appropriate motor behavior. After that, the autonomous stage will be reached in which the learner knows how to execute a task and does not have to think about the procedure, which may help the learner to concentrate on other aspects or the next part of the procedure [11]. Early stages of learning technical skills can be acquired outside the operating room using surgical simulation to allow trainees to focus on more complex issues when performing in theater [11]. Therefore, a multitude of technical learning tools may help for this purpose, such as surgical simulation using simple trainers, animal models, or an increasing amount of modern simulators using VR or augmented reality (AR).

### Simulation training

To train technical skills in open surgery as well as arthroscopic or laparoscopic surgery, different skill sets are necessary. These skills have to be learned and at best trained by repetitively performing the same procedure. Because of shortened working hours, cost intensity, and patient safety issues, trainees often cannot achieve the required expertise when only performing these procedures in the operating room. Therefore, the simulation of surgical procedures has become very important and advocated as a patient safety issue in surgical education [10], although there is evidence that supervised surgery performed by residents has no negative impact on the outcome of the patient [17], [18], [19]. Wojcik et al. stated in a pilot study that a minor surgery clinic run by residents may improve operative autonomy and still is safe for the patients [20]. Also, the often discussed exceeding operative time due to a procedure performed by a resident has been found comparable to an attendings’ performance [21].

The rapid technological development led to novel forms of surgical simulation in settings similar to real clinical settings [22], allowing surgeons to perform and train procedures in open surgery on synthetic models, human cadavers or living animals, in VR and AR. There are numerous advantages of the use of simulations. The trainee may learn surgical procedures and techniques step-by-step in a comforting surrounding and mistakes will not result in harm to the patient. Also, simulation allows an accurate assessment of the surgeon’s technical skills, which has greater validity than the assessment in theater [7]. Pantelidis et al. showed that the same level of expertise can be provided by either in vivo simulation or dry-lab simulation for fundamental laparoscopic surgery [23]. This may reduce the usage of animals to train surgeons. However, some procedures can only be simulated using in vivo simulation models, especially to train complications such as bleeding, which cannot be provided by common simulators. For this purpose, VR and AR are evolving in surgical education.

### Open surgical simulation

Increasing minimally invasive approaches result in a decreasing experience for open surgery in resident training. This may result in potentially critical situations if an operation has to be converted due to an adverse event either as a primary problem or because of a complication. Fonseca et al. analyzed the current usage of open surgery simulation in residency training and found that only a few studies focused on the use of simulation in the training of open surgical skills [24]. Thirty-one studies were found devoted to open surgical simulation [24]. However, the objectives varied from specific (evaluation of the learning curve) to general (evaluation of technical skill assessment tools) skills [24]. Although most studies used validated assessment tools, a large number of studies relied on self-assessment or course efficiency [24]. Using low-fidelity bench models (e.g. bananas and synthetic skin tissue) provide a beneficial tool for junior residents in basic surgical skills [9]. However, these cannot be used for specific surgical skills that are required by more advanced residents. Therefore, high-fidelity simulations are necessary, which can also improve junior residents’ skills [24]. Anastakis et al. showed that bench model training is transferable to the human model (cadaveric), yet no study analyzed the transfer to the operating room thus far. However, most authors proposed that these simulation models are helpful in theater as well [24]. Fonseca et al. recommended that these simulation models should focus on gaining experience for the identification of open anatomy, achieving adequate intraoperative exposure, performing surgical dissections efficiently and safely, and controlling major bleeding issues [24]. These high-fidelity simulation models are usually very expensive, which is why multi-institutional collaborations or the availability in workshops are often necessary [24]. However, simulation training in open surgery offers the opportunity to acquire competencies, which are required especially in emergency situations, although the transfer of skills to the operating room has to be validated in further studies.

### VR in surgical education

The use of VR in surgical education has been mainly described for laparoscopic and arthroscopic surgery [14]. These devices allow the training from basic handling skills in laparoscopy or arthroscopy to complete surgical procedures. The main advantage of these systems is that most VR simulators have a training program already implemented, which allows the trainee to perform the procedures with or without supervision by a trainer for further instructions. This reduces personal costs in the usage of these simulators as long as trainees have access to these systems. Although these technical skills are already implemented in surgical training programs in the United Kingdom, the access to such VR simulators differs locally (70% in Scotland to 30% in East England) [25]. However, there are no such data about the availability of these simulators in Germany, Austria, or Switzerland. Using VR simulators allow the measurement of precision and accuracy as well as error rates, which can be calculated easily [11], [26]. These high-fidelity VR simulators enable the training of complex procedures such as colonoscopy, carotid artery stenting, or rotator cuff reconstruction [11]. Alaker et al. reviewed 24 randomized controlled studies that compared VR versus “no training”. These studies analyzed error scores, OSATS, GOALS, GRS, distance movement, and accuracy, which all were significantly superior with VR training compared to no training [27]. Alaker et al. further stated that VR training, under supervision with prompt instructions and feedback and the use of haptic feedback, has proven to be the most effective way [27].

### Human factors in surgical education

The analysis of aviation accidents showed that the role of human factors was accountable for preventable critical errors [14], [28]. In surgical care, the awareness of patient safety and errors related to human factors increased in the last years but is still a minor field of research and financial resources. Especially, disruptions in the flow of an operation, teamwork, and communication failures contribute significantly to such adverse events [28]. Of course, the occurrence of human error is unavoidable, but some procedures can be better designed to prevent or detect these errors before the patient gets harmed. In times of an increasing work load and decreasing staff, the team of surgeons, nurses, and physical therapists has to be trained to work together and to achieve a better quality of patient care. However, only limited literature is available about training human factors in surgical care, although some training concepts already exist, such as the “Interpersonal Competence Course” in Germany [29] or the “Human Factors Training Course for Surgical Core Trainees” at the Severn Deanery School of Surgery in the United Kingdom [28], which already showed an increase of situational awareness or communication skills. However, the use of human factor courses is not implemented in many curricula thus far. The assessment of human factors can be performed using tools such as Surgeon Leadership Inventory [30] to analyze maintaining standards, managing resources, making decisions, directing, training, supporting others, communicating, and coping with pressure as nontechnical surgical skills (Table 2) [30]. Novel concepts adapted from programs of the aviation industry seem to enhance better patient care, although, for surgery, no long-term data are available at the current time point.

**Table 2:** Surgical leadership competency describes which competencies are required by a surgeon and how these are being taught by the teacher.

Surgeon leadership competency	
I	Maintaining standards – safety and quality of the procedure
II	Making decisions – choosing a solution to a problem
III	Managing resources – assigning resources depending on the situation
IV	Directing – giving clear instructions and stating expectations
V	Training – instructing and coaching team members according to the goals of the task
VI	Supporting others – offering assistance where appropriate
VII	Communicating – speaking appropriately for the situation
VIII	Coping with presssure – showing flexibility and changing in plans if necessary

## Discussion

Since the first residency programs for surgical training were introduced in Germany in the late 1880s and adopted by William Halsted in 1889 [31], surgical education has evolved from a sheer volume of exposure to structured curricula, however, surgical training programs still vary worldwide. Only sparse data are available comparing international surgical education programs for their structure and the surgical expertise after finishing the residency program. In some surgical disciplines, comprehensive surgical simulation curricula were established based on multiple studies. However, these curricula often are not mandatory, which result in published curricula (e.g. The Cardiac Surgical Society - TDSA published a 178-page curriculum that is available for free [32]) not used by teaching hospitals. Although a study, financed by the U.S. Government, analyzed the TDSA’s curriculum for its usefulness, the overall adaption rate is low because costs and resources impede broad implementation [32]. Governmental regulation does not require simulation training, and financial reimbursement for surgical training is low or does not exist. Thus, there is no promotion for excellent, good, or bad surgical education by governmental payment; for example, the German DRG does not distinguish between senior surgeons and residents, which results in decreased surgical procedures performed by residents because of increasing commercial interests of surgical procedures.

After reviewing the literature, we can only estimate that structured curricula are helpful because they provide assessments after defined time points, which allows the resident and the faculty to address problems. We can only estimate that this would also help to increase the relevance of surgical education for financing such programs by hospital administrations. In contrast, structured curricula reduce the flexibility and individualized training provided by the trainers; also, structured curricula are vulnerable to a lack of personnel, which will be a growing concern due to the decreasing number of medical professionals in the following years [4], [16].

## Conclusion

Surgical education is still an evolving field of research and novel methods for surgical education will change current surgical curricula for future modern surgeons. However, it is necessary that surgical societies increase their commitment to provide the missing link between research and implementation of these educational tools in structured education programs in the future.

## Supplementary Material

The article (iss-2018-0026) offers reviewer assessments as supplementary material.
