# Supplementary material for: Evidence in surgical training – a review
Source: Innov Surg Sci. 2019 Apr 22;4(1):7–13. doi: 10.1515/iss-2018-0026 (PMC6754061; doi:10.1515/iss-2018-0026)
Supplement: Supplementary file 1 [file iss-4-20180026-s001.pdf]

Review

Tobias Fritz*, Niklas Stachel and Benedikt J. Braun

Evidence in surgical training – a review

https://doi.org/10.1515/iss-2018-0026
Received September 10, 2019; accepted March 21, 2019

***Corresponding author: Tobias Fritz,** Department of Trauma, Hand and Reconstructive Surgery, Saarland University Medical Center, Kirrbergerstr. 1, 66421 Homburg/Saar, Germany, E-mail: tobias.fritz@uks.eu

Reviewers' Comments to Original Submission

Reviewer 1: Axel Haverich

Oct 16, 2018

**Reviewer Recommendation Term:**	Accept
**Overall Reviewer Manuscript Rating:**	90

**Custom Review Questions**	**Response**
Is the subject area appropriate for you?	5 - High/Yes
Does the title clearly reflect the paper's content?	4
Does the abstract clearly reflect the paper's content?	5 - High/Yes
Do the keywords clearly reflect the paper's content?	5 - High/Yes
Does the introduction present the problem clearly?	N/A
Are the results/conclusions justified?	4
How comprehensive and up-to-date is the subject matter presented?	4
How adequate is the data presentation?	4
Are units and terminology used correctly?	4
Is the number of cases adequate?	N/A
Are the experimental methods/clinical studies adequate?	N/A
Is the length appropriate in relation to the content?	4
Does the reader get new insights from the article?	4
Please rate the practical significance.	4
Please rate the accuracy of methods.	N/A
Please rate the statistical evaluation and quality control.	N/A
Please rate the appropriateness of the figures and tables.	3
Please rate the appropriateness of the references.	4
Please evaluate the writing style and use of language.	4
Please judge the overall scientific quality of the manuscript.	4
Are you willing to review the revision of this manuscript?	Yes

Comments to Authors:
Dear Editors, Dear Authors,
the manuscript titled "Evidence in Surgical Education" is an important contribution to the surgical field of training and education. We suggest publication and have the following comments:

Line 48-53: Although one can guess it from the text, I suggest to emphasize two important facts about the German surgical training system: 1. The official trainer of the institution (not necessarily the senior surgeon, who trains the residents) certifies that the resident has acquired the necessary knowledge and skills and performed the demanded procedures. The content of the certification is not checked by

Open Access. © 2019 Fritz T., et al., published by De Gruyter. This work is licensed under the Creative Commons Attribution 4.0 Public License.

an independent source. 2. Surgical quality is not part of this certfication to distinguish the good from the average surgeon. A practical test (e.g. in index procedures on a simulator) is not performed.

Line 263-: The content of the discussion is in many aspects redundant to the initial chapters of the manuscript. I suggest to incorporate the additional content of the discussion in the sub-chapters and to use the content of "conclusion" as the discussion chapter.

There are two aspects that in our view should be added to the discussion and for future perspective:
1. In some disciplines, comprehensive surgical simulation curricula were established based on multiple studies. Their positive effect can be considered as "proven" and are especially needed in disciplines with complex equipment (e.g. robotic surgery, videoscopic surgery, microsurgery). For cardiac surgery, for example, the TSDA published a 178 page long curriculum (https://www.tsda.org/wp-content/uploads/2016/01/Cardiac-Surgery-Simulation-Curriculum-TSDA.pdf), that is available for free but most likely not used in most teaching centers in the US. An US-study financed by governmental (!) authorities (Mokadam NA, et al. The Annals of thoracic surgery. 2017;103(1):322-8.) established its usefulness. Nevertheless, the overall adoption rate is low, because costs and resources impede broad implementation. Governmental regulations do not require simulation training and financial reimbursement for surgical training is low or does not exist. The same is true for the DRG-system in Germany that does not promote good (or even any) surgical education.

2. To my knowledge, there are no national requirements in Germany to finish simulations courses as part of a surgical curriculum or as prerequisite for board certification. In Switzerland simulation courses are part of the residency curriculum for vascular surgery, for example. Making simulation courses mandatory is the easiest way to increase the adoption rate in teaching hospitals. However, this leads to another problem: The political lobby to improve surgical education is small, although the interest to improve patient care is high. What is commonplace in aviation (high regulatory standards for training to ensure quality and safety, e.g. including regular simulation courses for fully certified personnel) is not used in surgery. Surgical education in many countries currently depends on dedicated and motivated individuals that use the small resources they have at hand to teach the next generation surgeons at their institution. Although I hate to advocate more regulation in our line of work: Regulations and proper payment are probably the only way to increase the overall interest in research on surgical education and to increase available evidence on effective surgical teaching and training.

Reviewer 2: anonymous

Mar 11, 2019

**Reviewer Recommendation Term:**	Accept with Minor Revision
**Overall Reviewer Manuscript Rating:**	75
**Custom Review Questions**	**Response**
Is the subject area appropriate for you?	4
Does the title clearly reflect the paper's content?	4
Does the abstract clearly reflect the paper's content?	4
Do the keywords clearly reflect the paper's content?	4
Does the introduction present the problem clearly?	4
Are the results/conclusions justified?	3
How comprehensive and up-to-date is the subject matter presented?	5 - High/Yes
How adequate is the data presentation?	2
Are units and terminology used correctly?	N/A
Is the number of cases adequate?	N/A
Are the experimental methods/clinical studies adequate?	N/A
Is the length appropriate in relation to the content?	2
Does the reader get new insights from the article?	4
Please rate the practical significance.	4
Please rate the accuracy of methods.	N/A
Please rate the statistical evaluation and quality control.	N/A
Please rate the appropriateness of the figures and tables.	1 - Low/No
Please rate the appropriateness of the references.	5 - High/Yes
Please evaluate the writing style and use of language.	2
Please judge the overall scientific quality of the manuscript.	3
Are you willing to review the revision of this manuscript?	Yes

Comments to Authors:

The review is dealing with a very interesting topic. Surgical education is very important. Today, various teaching tools with modern learning platform are available.
The language is sometimes difficult to understand and needs minor revision.
In summary very interesting report which should be published in Innovative Surgical Sciences.

Authors' Response to Reviewer Comments

Mar 16, 2019

Reviewer #1:

Thank you for your suggestions.

Line 48-53 – I updated the segment.

Line 273 and following – I updated the segment as suggested and implemented the additional information in the previous chapters.
I also added your statements into the manuscript.
I think the task of a promotional system for excellent trainers is very important, but a very "hot" political topic, without any scientific data and evidence, but maybe this could be part of a future study.
I tried to implement the statements and I hope you are ok with it.

Reviewer #2

Thank you for your kind words!
I updated the manuscript and I hope the language is better to understand now.
